# Rapid assessment of bovine spongiform encephalopathy prion inactivation by heat treatment in yellow grease produced in the industrial manufacturing process of meat and bone meals

**DOI:** 10.1186/1746-6148-9-134

**Published:** 2013-07-09

**Authors:** Miyako Yoshioka, Yuichi Matsuura, Hiroyuki Okada, Noriko Shimozaki, Tomoaki Yamamura, Yuichi Murayama, Takashi Yokoyama, Shirou Mohri

**Affiliations:** 1 Prion Disease Research Center, National Institute of Animal Health, 3-1-5 Kannondai, Tsukuba, Ibaraki 305-0856, Japan; 2 Research Area of Pathology and Pathophysiology, National Institute of Animal Health, 3-1-5 Kannondai, Tsukuba, Ibaraki 305-0856, Japan

**Keywords:** Prion inactivation, Bovine spongiform encephalopathy, Meat and bone meal, Yellow grease, Infectivity, Protein misfolding cyclic amplification

## Abstract

**Background:**

Prions, infectious agents associated with transmissible spongiform encephalopathy, are primarily composed of the misfolded and pathogenic form (PrP^Sc^) of the host-encoded prion protein. Because PrP^Sc^ retains infectivity after undergoing routine sterilizing processes, the cause of bovine spongiform encephalopathy (BSE) outbreaks are suspected to be feeding cattle meat and bone meals (MBMs) contaminated with the prion. To assess the validity of prion inactivation by heat treatment in yellow grease, which is produced in the industrial manufacturing process of MBMs, we pooled, homogenized, and heat treated the spinal cords of BSE-infected cows under various experimental conditions.

**Results:**

Prion inactivation was analyzed quantitatively in terms of the infectivity and PrP^Sc^ of the treated samples. Following treatment at 140°C for 1 h, infectivity was reduced to 1/35 of that of the untreated samples. Treatment at 180°C for 3 h was required to reduce infectivity. However, PrP^Sc^ was detected in all heat-treated samples by using the protein misfolding cyclic amplification (PMCA) technique, which amplifies PrP^Sc^*in vitro*. Quantitative analysis of the inactivation efficiency of BSE PrP^Sc^ was possible with the introduction of the PMCA_50_, which is the dilution ratio of 10% homogenate needed to yield 50% positivity for PrP^Sc^ in amplified samples.

**Conclusions:**

Log PMCA_50_ exhibited a strong linear correlation with the transmission rate in the bioassay; infectivity was no longer detected when the log PMCA_50_ of the inoculated sample was reduced to 1.75. The quantitative PMCA assay may be useful for safety evaluation for recycling and effective utilization of MBMs as an organic resource.

## Background

Transmissible spongiform encephalopathies (TSEs), including scrapie in sheep and goats, chronic wasting disease (CWD) in deer and elk, bovine spongiform encephalopathy (BSE) in cattle, and Creutzfeldt–Jakob disease (CJD) in humans, are infectious and fatal neurodegenerative diseases [1]. Proteinaceous infectious agents called prions are thought to be responsible for TSEs, which are characterized by the accumulation of the pathogenic form of prion protein (PrP^Sc^) in the nervous tissues of infected subjects [2,3]. PrP^Sc^ is a conformational isoform of the normal cellular prion protein (PrP^C^), which is rich in beta-sheet structures, insoluble in mild detergents, and resistant to protease digestion [4,5].

Because prions retain infectivity after undergoing routine sterilization processes [6], contaminated meat and bone meals (MBMs) are suspected to be the source of BSE infection [7,8]. MBMs are manufactured through a multi-step process involving the crushing of carcasses in a pre-breaker, heating at 120°C–140°C in yellow grease (lower-quality grades of tallow) in a cooker, and degreasing from solid material by an oil separator. To determine BSE prion inactivation during the manufacturing process of MBMs, industrial processes were replicated on a pilot scale by using BSE-infected brains, and the infectivity of processed materials in each step was investigated in detail [9]. However, in reality, processing conditions for MBMs differ among rendering houses producing commercial MBMs. Since the efficiency of prion inactivation could be influenced by various factors such as treatment temperature, time, steam pressure in the cooker, size, water and fat contents of carcasses [10-13], it is difficult to identify the risks attributable to specific processing conditions. Furthermore, PrP^Sc^ retained in the manufacturing process of MBMs remains to be elucidated.

The governments of many countries prohibited the feeding of bovine MBMs following the feeding ban on MBMs in the United Kingdom. A prion detection method with high sensitivity and high accuracy must be developed so that MBMs can be used safely in the future. In addition, BSE prion is more resistant to physical and chemical treatments than are scrapie and CJD prions [14]. Therefore, experiments using BSE-infected materials are essential for the assessment of BSE prion inactivation as they can be considered a worst case among prions. In recent years, it has become possible to perform *in vitro* amplification of PrP^Sc^ derived from various animals [15-21] by using protein misfolding cyclic amplification (PMCA) [22]. We developed an ultrasensitive method for BSE PrP^Sc^ detection using potassium dextran sulfate (DSP) [20]. The PMCA technique can also be used to quantitatively assess scrapie PrP^Sc^[23-25], and our PMCA method can be applied as an effective test for the assessment of prion inactivation by monitoring residual BSE PrP^Sc^[26]. In the present study, we investigated efficiency of BSE prion inactivation following heat treatment in yellow grease by bioassay and quantitative PMCA.

## Results

### Infectivity of heat-treated homogenates

Long-term follow-up confirmed infectivity in the mice intracerebrally inoculated with up to a 10^–5^ dilution of the 10% homogenate of the pooled spinal cords (Table 1). PrP^Sc^ accumulation was confirmed in the brains of the diseased mice by western blotting and histopathological analysis (data not shown). The infectious titer of the homogenate was estimated to be 10^6.7^ LD_50_ per gram. A strong linear correlation (r = 0.99) between the incubation times and dilution ratios of the inoculated homogenate was observed in mice inoculated with up to a 10^–3^ dilution. Some mice inoculated with 10^–4^ and 10^–5^ diluted samples developed the disease after similar prolonged survival times (735 or 736 days). In the extreme dilution range, lower rate of transmission and prolonged incubation time are generally observed in the mice intracerebrally inoculated with prion-infected brain homogenates. Since PrP^Sc^ tends to aggregate, these phenomena may be due to the near-absence of PrP^Sc^ which would have been almost completely diluted out.

**Table 1:** Mean incubation time of TgBoPrP mice following intracerebral inoculation of titrated bovine spongiform encephalopathy (BSE)-infected spinal cord homogenate

**10% Homogenate dilution**	**Transmission rate (diseased/total)**	**Mean incubation time ± SD (days)**
10^0^	100% (7/7)	242 ± 14
10^–1^	100% (7/7)	279 ± 12
10^–2^	100% (5/5)	322 ± 42
10^–3^	100% (6/6)	367 ± 53
10^–4^	33% (2/6)	736, 736, >790
10^–5^	17% (1/6)	735, >790
10^–6^	0% (0/6)	>790

Table 2 shows the effect of various heat treatments in yellow grease on the BSE-infected spinal cord homogenates. All mice inoculated with samples treated at 140°C for 1 h died after an average of 304 days. The infectivity was reduced to approximately 1/35 (log reduction = 1.54) following the heat treatment. When the samples subjected to temperatures above 140°C were used, 100% (180°C for 1 h) and 67% (160°C for 1 h) of the mice developed the disease after prolonged average survival times. Regarding the treatments for 3 h, infectivity was still detected in some mice inoculated with the samples treated at 140°C or 160°C. Because the incubation times of these diseased mice were beyond the range of application of the regression line obtained using the titrated BSE-infected homogenates, the log reduction of infectivity in each sample was estimated to be more than 3.0. Meanwhile, mice inoculated with samples treated at 180°C for 3 h did not exhibit disease onset 790 days after inoculation.

**Table 2:** Effects of various heat treatments in yellow grease on BSE-infected spinal cord homogenates

**Temperature**	**Time (h)**	**Transmission rate (diseased/total)**	**Mean incubation time ± SD (days)**	**Log reduction of infectivity**	**Log reduction of PMCA**_**50**_
140°C	1	100% (6/6)	304 ± 13^*,‡^	1.54	2.75
	3	83% (5/6)	382 ± 64, >790	>3.0	4.0
160°C	1	67% (4/6)	471 ± 80^*,†^, >790	>3.0	3.5
	3	17% (1/6)	514, >790	>3.0	6.0
180°C	1	100% (6/6)	380 ± 25^†,‡^	>3.0	3.25
	3	0% (0/6)	>790	>3.0	6.75

^*,†,‡^ Significant differences (*: *p* < 0.01; †, ‡: *p* < 0.05) were observed among mice with respect to the mean incubation times as indicated by identical superscript character.

### PrP^Sc^ detection by PMCA

**Figure 1:**
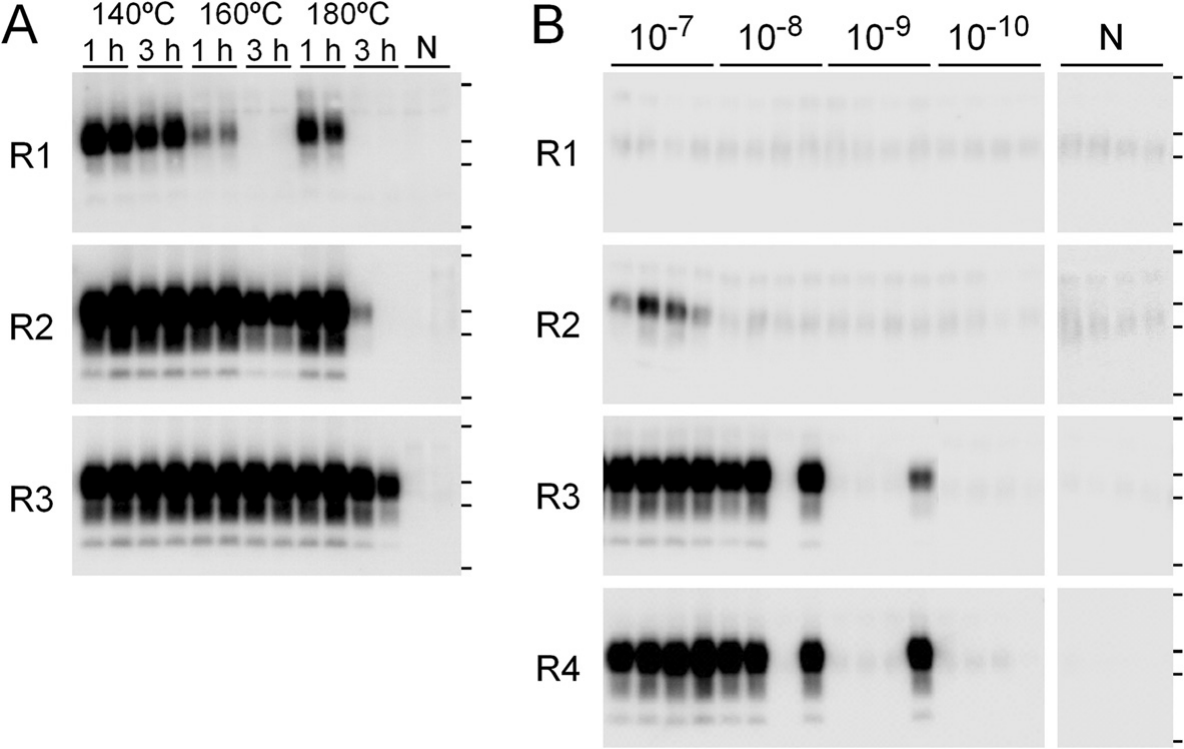
**Detection of bovine spongiform encephalopathy (BSE) PrP**^**Sc **^**by serial potassium dextran sulfate-protein misfolding cyclic amplification. (A)** Homogenates (10%) of BSE-infected spinal cords treated in yellow grease at 140°C–180°C for 1 or 3 h were diluted 10^–1^ with the PrP^C^ substrate and amplified by serial PMCA. Duplicate samples were analyzed after each round (R1–R4) of amplification by western blotting after digestion with proteinase K. The lanes labeled “N” are samples in which only the PrP^C^ substrate was treated in the same manner. Horizontal lines indicate the positions of molecular-weight markers corresponding to 37, 25, 20, and 15 kDa. **(B)** Homogenates (10%) of the heat-untreated BSE-infected spinal cords were diluted 10^–7^ to 10^–10^ with the PrP^C^ substrate and amplified in four tubes by serial PMCA.

Figure 1a illustrates the results of the amplification of the samples subjected to the grease-heating method. No PrP^Sc^ signals were detected in the heat-treated samples by western blotting before amplification (data not shown). After one round of amplification, PrP^Sc^ signals were detected in the samples treated at 140°C–180°C for 1 h and at 140°C for 3 h. PrP^Sc^ signals were also detected in both duplicate samples treated at 160°C and 180°C for 3 h after two or three rounds of amplification. In the samples treated at 180°C for 3 h, trace amounts of PrP^Sc^ remained after the treatment, although infectivity was not detected in the bioassay.

### Quantitative analysis of PrP^Sc^

Figure 1b shows the results of the amplification of each diluted sample of untreated BSE-infected spinal cord homogenate. PrP^Sc^ present in 10^–7^ dilution of the infected homogenate was detected in all tubes after three rounds of amplification. PrP^Sc^ signals were detected in three of the 10^–8^ and one of the 10^–9^ dilutions after three rounds of amplification. However, no additional tubes became positive for PrP^Sc^ in these dilutions after four rounds of amplification. No signals were detected in the more extreme dilution ranges even after four rounds of amplification. Thus, the PMCA_50_ of the 10% homogenate was calculated to be 10^8.5^ units on the basis of the results obtained from the fourth round of amplification.

**Figure 2:**
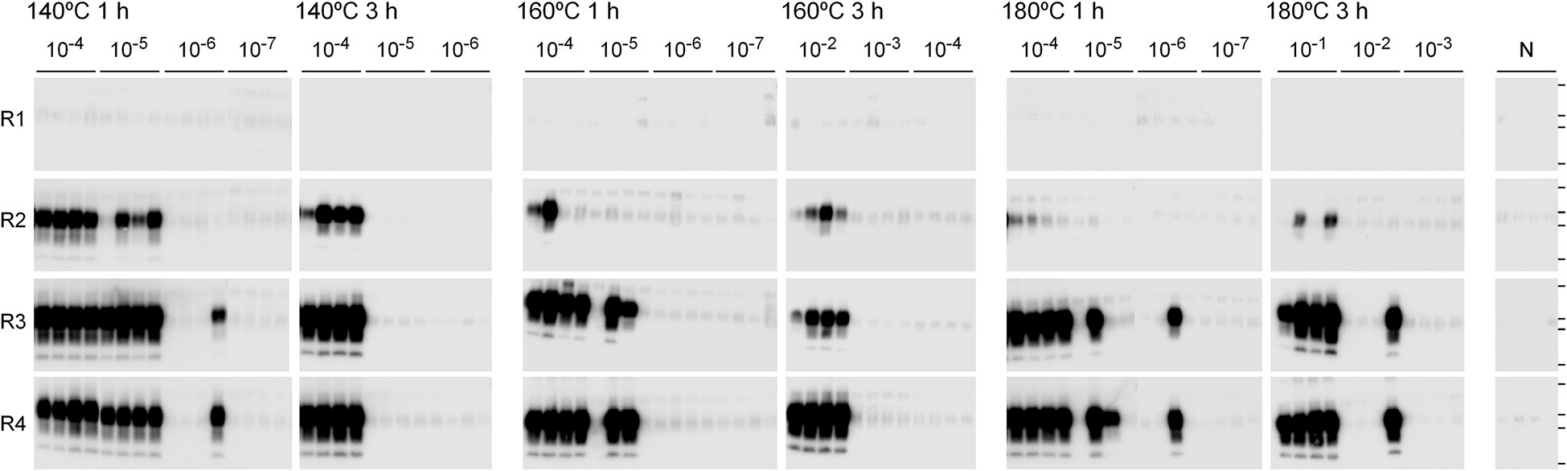
**Quantitative analysis of bovine spongiform encephalopathy (BSE) PrP**^**Sc **^**in heat-treated samples.** Homogenates (10%) of BSE-infected spinal cords treated in yellow grease at 140–180°C for 1 or 3 h were diluted with the PrP^C^ substrate and amplified by serial dextran sulfate-protein misfolding cyclic amplification. The dilution ratios examined in each sample are indicated. Quadruplicate samples were analyzed after each round (R1–R4) of amplification by western blotting after digestion with proteinase K. The lanes labeled “N” are samples in which only the PrP^C^ substrate was treated in the same manner. Horizontal lines indicate the positions of molecular-weight markers corresponding to 37, 25, 20, and 15 kDa.

To evaluate the PrP^Sc^ inactivation efficiency of each heat treatment, we estimated the PMCA_50_ from the results obtained at the fourth round of amplification of serial 10-fold dilutions of heat-treated samples. Serial PMCA was sufficiently sensitive to detect PrP^Sc^ in these diluted samples (Figure 2). The log reduction of PMCA_50_ values of the heat-treated samples are shown in Table 2. Regarding the treatments for 1 h, PrP^Sc^ inactivation appeared to be most efficient in the samples treated at 160°C. This finding is concordant with the observations of partial transmission of infectivity (67%) in the mice inoculated with this sample and prolonged incubation times of the diseased mice. Log PMCA_50_ decreased with extended heat treatment time: although 180°C for 3 h was the most effective treatment, it was unable to completely inactivate the proportion of PrP^Sc^ that is amplifiable by serial PMCA.

**Figure 3:**
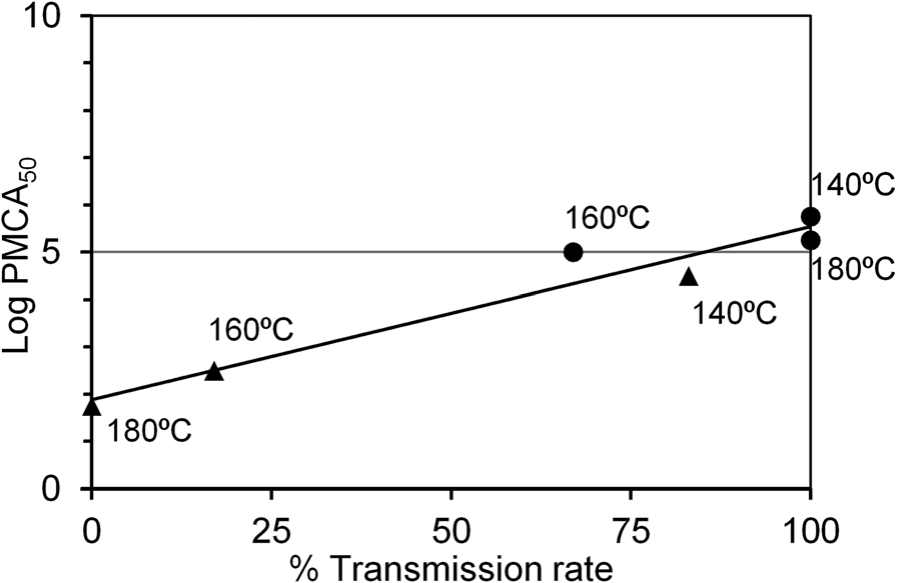
**Relationship between the log PMCA**_**50 **_**and transmission rate in the bioassay.** A strong linear relationship (r = 0.97) was observed in the samples treated at 140°C–180°C for 1 h (●) and 3 h (▲).

Figure 3 shows the relationships between the transmission rate in the bioassay and the log PMCA_50_ values of the inoculated samples. A strong linear correlation (r = 0.97) was observed between the log PMCA_50_ values and transmission rate. When the log PMCA_50_ exceeded 5.25, the transmission rate in the bioassay reached 100% as observed in the mice inoculated with samples treated at 140°C or 180°h for 1 h. Infectivity was not detected in the mice when the log PMCA_50_ of the inoculated sample was reduced to 1.75.

## Discussion

In this study, BSE prion inactivation was analyzed quantitatively in terms of infectivity and the PrP^Sc^ contents of the samples after heat treatment in yellow grease. Following treatment at 140°C for 1 h, which is the heat treatment condition generally used for carcasses in rendering houses in Japan, the infectivity of the BSE-infected spinal cord homogenate was reduced to at most 1/35 of that of the untreated control samples; furthermore, PrP^Sc^ retained its capability for *in vitro* propagation. Because carcasses are usually heat treated in closed cookers, prions are affected by the steam pressure from the water contained in the carcass. If a sufficient amount of water is present in the carcass, prion inactivation may proceed more efficiently in the cooker than under atmospheric pressure. However, some degree of BSE infectivity was still detected after autoclaving at 133°C in spiked raw materials with high infectivity levels [26]. Furthermore, the precise effects of high-pressure steam on carcasses submerged in yellow grease are not known. Therefore, high-risk materials such as brains and spinal cord should be excluded from the rendering process for effective inactivation of BSE prion.

We previously examined residual infectivity and PrP^Sc^ after heat treatment of scrapie-infected hamster brains under various experimental conditions [27]. The PMCA results were concordant with bioassay results. However, BSE PrP^Sc^ was detected in the samples treated at 180°C for 3 h, although infectivity was not detected in the bioassay. There are several possible explanations for this discrepancy between infectivity and PrP^Sc^ occurrence. For example, BSE PrP^Sc^ might contain various forms of PrP^Sc^ with different amplification properties and infectivity, and a PMCA-compatible form of PrP^Sc^ with low or no infectivity might predominate after heat treatment and be maintained over other forms throughout the amplification process. However, in the present study, the log PMCA_50_ values were strongly correlated with the transmission rate in the bioassay (Figure 3), suggesting that such PMCA-compatible but less-infectious PrP^Sc^ was not selectively amplified *in vitro*.

In our previous paper, we demonstrated our amplification system was highly sensitive and accurate, and no spontaneous generation of PrP^Sc^ was observed in the amplification of various kind of samples derived from uninfected animals [20]. Determination of PMCA_50_ based on quadruplicate amplification was also done in our previous study [26], and we confirmed that similar PMCA_50_ values (around 10^11^ per gram) were obtained in two independent studies. In the present study, the PMCA_50_ of the BSE-infected spinal cords was estimated to be 10^11.6^ per gram, which is approximately 80,000-fold greater than the corresponding intracerebral LD_50_ per gram (10^6.7^) determined by the bioassay. The PMCA_50_/LD_50_ per gram of BSE prion was considerably higher than those of scrapie prion strains (160–4000 fold) [25]. If this ratio reflects the number of PrP^Sc^ particles that compose an infectious unit of prions, more PrP^Sc^ particles might participate in an infectious unit of BSE prions; moreover, such a large mass of PrP^Sc^ particles might be processed into several smaller ones with lower infectivity *in vivo*.

**Figure 4:**
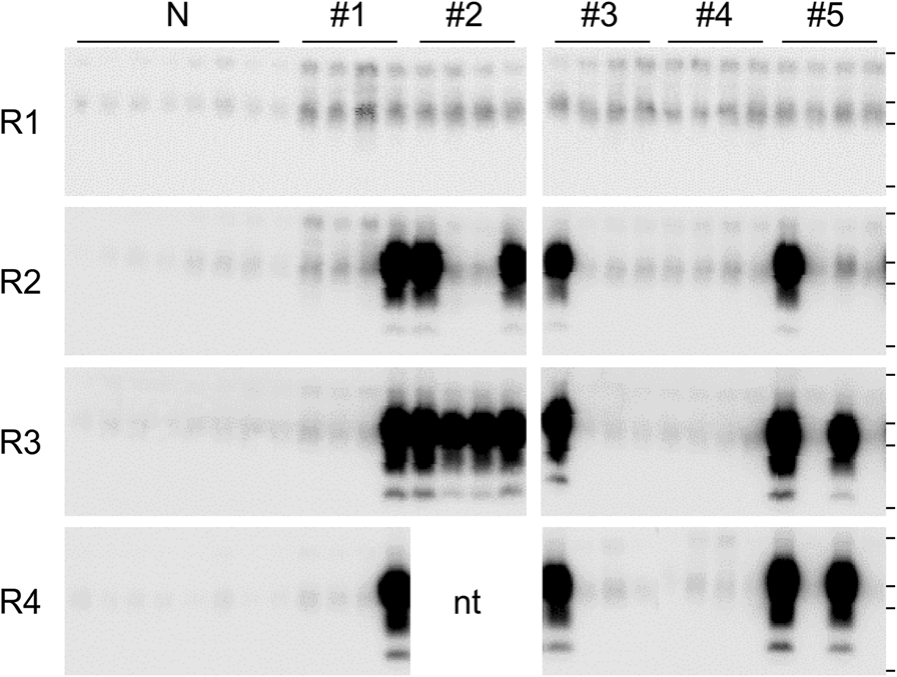
**Detection of PrP**^**Sc **^**in brains of asymptomatic mice.** A 10% brain homogenate from five (#1-#5) of six asymptomatic mice inoculated with 10^–6^ dilution of infected homogenate (Table 1) was prepared, and amplified by serial PMCA. Quadruplicate samples were analyzed after each round (R1–R4) of amplification by western blotting after digestion with proteinase K. One (#1 and #3), two (#5) and four (#2) of the quadruplicate samples were found to be positive for PrP^Sc^ after three or four rounds of amplification. No PrP^Sc^ signal was detected in #4 mouse. The eight lanes labeled “N” are samples in which only the PrP^C^ substrate was treated in the same manner. Horizontal lines indicate the positions of molecular-weight markers corresponding to 37, 25, 20, and 15 kDa. nt: not tested.

Alternatively, PrP^Sc^ accumulation might proceed in animals inoculated with PrP^Sc^ when the PrP^Sc^ concentration is below a specific cut-off, but the animals might not develop the disease within their lifetimes. Actually, clinically asymptomatic infections are known as the subclinical infection stage [28-30]. In the present study, we examined PrP^Sc^ in brains of asymptomatic mice inoculated with titrated BSE-infected homogenate, and PrP^Sc^ was found at various levels in four of five mice inoculated with 10^–6^ dilution of the infected homogenate (Figure 4). Therefore, pathogenicity might be detected by serial transmission in animals as in the case of serial PMCA. If so, the detection sensitivity of the bioassay used in the present study may not be sufficiently high for proper safety evaluation, because ecycling of BSE-infected bovine tissues possibly augments the concentration of PrP^Sc^ in commercial MBMs if the carcasses contain infinitesimal amounts of prion.

Another aspect of heat treatment in yellow grease is that higher-temperature treatments do not necessarily inactivate BSE prion more effectively. In the case of heat treatment for 1 h, the results of both the bioassay and PMCA indicate that BSE prion inactivation proceeded more effectively with treatment at 160°C rather than at 180°C. Samples treated at 180°C were dark brown, suggesting that the surface was scorched during treatment. In such high-temperature conditions, thermal conduction may be inhibited by scorching of the sample periphery, consequently requiring longer treatment time to reach thermal equilibrium in the sample. Extension of the treatment time to 3 h was actually necessary for the loss of infectivity. However, further studies are needed to confirm the above possibility.

## Conclusions

In this study, we demonstrated that heat treatment at 180°C for 3 h is required for the loss of infectivity of BSE prion in grease heating in our experimental conditions. Furthermore, BSE PrP^Sc^ retains amplification ability even after such a treatment. The inactivation efficiency of BSE PrP^Sc^ could be quantitatively analyzed with the introduction of the PMCA_50_, which is strongly correlated with the transmission rate in the bioassay. The serial PMCA technique is more practical and less time consuming than bioassays, and may be applicable for monitoring residual PrP^Sc^ in the other steps of the manufacturing of MBMs and useful for safety evaluation for recycling and effective utilization of MBMs as an organic resource.

## Methods

### Experimental heat treatment procedure

All animal experiments were approved by the Animal Care and Use Committee of the National Institute of Animal Health (approval IDs: 450 and 08-008) in accordance with the Guidelines for Animal Transmissible Spongiform Encephalopathy Experiments of the Ministry of Agriculture, Forestry, and Fisheries of Japan. Spinal cords were obtained from four cows experimentally inoculated with BSE at the terminal stage of the disease. The infected materials were pooled and homogenized using a blender. Pure homogenate (0.5 g) was placed on a strip of aluminum foil (2 cm × 2 cm) and stored at –80°C until further use. For use, the homogenate with the aluminum foil was thawed at room temperature and then immersed in 15 mL yellow grease preheated to 140°C, 160°C, or 180°C in a ceramic crucible by using an electric heating device (ND-M11, Nissin Rika, Tokyo, Japan). The yellow grease used was obtained from a rendering house in Japan. The crucible was covered, and a thermosensor was inserted through a hole in the cover to monitor the temperature of the yellow grease. The yellow grease was, then, kept for 1 or 3 h at the desired temperature. The homogenate sample firmly adhered to the surface of aluminum foil and was not broken into pieces during the heat treatment. After the treatment, the homogenate with the aluminum foil was removed from the yellow grease with tweezers and placed on a paper towel for absorption of the excess yellow grease. The weights of the homogenates were reduced to 60–70% of their original weights. The resultant materials were thoroughly crushed with a mortar, and suspended in PBS at 10% (w/v). Insoluble materials were separated by brief centrifugation, and aqueous fraction was stored at –80°C until further use.

### Bioassay

Infectivity titer using transgenic mice overexpressing bovine PrP^C^ is generally 100-1000 times higher than that using cows. Therefore, more accurate estimation of BSE infectivity is able to be conducted by using such mice. The heat-treated samples were injected intracerebrally into six Tg(BoPrP)4092HOZ/Prnp^0/0^ (TgBoPrP) transgenic mice (20 μL per mouse) overexpressing bovine PrP^C^[31]. To determine the infectivity titer, serial 10-fold dilutions of the 10% homogenate of the untreated spinal cords were prepared in PBS and injected intracerebrally into five to seven TgBoPrP mice (20 μL per mouse). After inoculation, the mice were evaluated daily for signs of infection. The lethal dose (LD_50_) was determined according to the 50% endpoint calculation method. Mean incubation times of the diseased mice were analyzed by one-way ANOVA and Tukey’s multiple comparison test.

### PMCA

Bovine PrP^Sc^ was amplified as described previously [20]. Briefly, the brains of TgBoPrP transgenic mice and PrP knockout (PrP^0/0^) mice were homogenized separately in PBS containing 1% Triton X-100 and 4 m mol L^–1^ EDTA. After centrifugation at 4500 × *g* for 5 min, the supernatants were mixed in PrP^0/0^/TgBoPrP (5:1). A mixture containing 0.5% DSP was used as the PrP^C^ substrate for PMCA.

The 10% homogenates of heat-treated samples were mixed at 1:9 with the PrP^C^ substrate (total volume, 100 μL) in electron beam-irradiated polystyrene tubes. Amplification was performed in duplicate with a fully automatic cross-ultrasonic protein-activating apparatus (Elestein 070-CPR, Elekon Science, Chiba, Japan), which has a capacity to generate high ultrasonic power (700 W). PMCA amplification was performed by 40 cycles of sonication (3-s pulse oscillations repeated 5 times at 1-s intervals), followed by incubation at 37°C for 1 h with agitation. For serial PMCA, 1:5 dilution of the PMCA product and subsequent amplification was repeated twice.

To evaluate the inactivation efficiency of BSE PrP^Sc^ by heat treatment, the PMCA_50_, which is the dilution ratio of the 10% homogenate needed to yield 50% PrP^Sc^ positivity for amplified samples, was determined. Serial 10-fold dilutions of the 10% homogenate of the heat-treated and untreated samples were prepared and mixed 1:9 with the PrP^C^ substrate (total volume, 80 μL) and amplified in electron beam-irradiated eight-strip polystyrene tubes (076-96, Elekon Science). Amplification was performed in quadruplicate using 40 cycles of sonication (pulse oscillation for 5 s, repeated 5 times at 1-s intervals), followed by incubation at 37°C for 1 h with agitation. For serial PMCA, 1:5 dilution of the amplified product and subsequent amplification was repeated 3 times. The PMCA_50_ was estimated from the results of the fourth round of amplification by using the 50% endpoint calculation method.

### Western blotting

The amplified samples (10 μL) were mixed with 10 μL proteinase K solution (100 μg mL^–1^) and incubated at 37°C for 1 h. The digested samples were mixed with 20 μL 2× SDS sample buffer and incubated at 100°C for 5 min. The samples were separated by SDS-PAGE and transferred onto a polyvinylidene fluoride membrane (Millipore, Bedford, MA). After the membrane was blocked, it was incubated for 30 min with a horseradish peroxidase (HRP)-conjugated T2 monoclonal antibody [32]. After washing, the blotted membrane was developed using the Luminata Forte Western HRP Substrate (Millipore) according to the manufacturer’s instructions. Chemiluminescence signals were analyzed with a Light Capture System (Atto, Tokyo Japan).

### Histopathological analysis

The left hemispheres of the brains were fixed in 10% buffered formalin for neuropathological analysis. Coronal brain sections were immersed in 98% formic acid to reduce infectivity and embedded in paraffin wax. Sections (4 μm thick) were cut and stained with hematoxylin and eosin, and analyzed immunehistochemically as described previously [20].

## Abbreviations

PrPSc: Pathogenic form of prion protein; BSE: Bovine spongiform encephalopathy; MBMs: Meat and bone meals; PMCA: Protein misfolding cyclic amplification; TSEs: Transmissible spongiform encephalopathies; CWD: Chronic wasting disease; CJD: Creutzfeldt–Jakob disease; PrPC: Normal cellular prion protein.

## Competing interests

The authors declare that they have no competing interests.

## Authors’ contributions

MY and YM (Murayama) designed and prepared the manuscript. MY, YM (Matsuura), HO and YM (Murayama) performed the experiments. NS and TY helped to perform the experiments. TY and SM supervised the study. All authors have read and approved the final manuscript.
